# SCOPING REVIEWS: A METHODOLOGY TO UNDERSTAND HEALTHY AGING AND IDENTIFY NEW AREAS FOR RESEARCH

**DOI:** 10.1093/geroni/igae098.0684

**Published:** 2024-12-31

**Authors:** Basia Belza

**Affiliations:** Chair

## Abstract

Scoping reviews is an approach to synthesize evidence. They are used to categorize literature in a field in terms of its nature, features, and volume. The purpose of this symposium is to provide an overview of how to conduct a scoping review and highlight examples of recent scoping reviews. The first presentation builds on the social determinants of health report by World Health Organization (WHO) and defines and categorizes approaches that have been implemented to achieve health equity among older adults. The second presentation is on social frailty which results from a lack of social resources and support and poses a significant threat to healthy aging by increasing the risk of adverse health outcomes. Creatively using a person-environment-occupation model, the third presentation categorizes the effectiveness of interventions into mealtime for people living with dementia who reside in care communities. Building on the growing body of literature that links benefits of music to health outcomes, the fourth presentation focuses on a review of the terminology used for music-based interventions. The fifth presentation focuses on identifying the outcomes of community-based physical activity programs for people with dementia, a group that frequently is missing from our study populations. The symposium will conclude with recommendations for areas that need improvement related to scoping reviews: 1) engaging stakeholders earlier and more often with scoping reviews to facilitate interpretation of results and effective knowledge translation, and 2) identifying ways to use the scoping review results as a decision-making tool.

